# Optimization, In Vitro and Ex Vivo Assessment of Nanotransferosome Gels Infused with a Methanolic Extract of *Solanum xanthocarpum* for the Topical Treatment of Psoriasis

**DOI:** 10.3390/gels10020119

**Published:** 2024-02-02

**Authors:** Nilanchala Sahu, Perwez Alam, Asad Ali, Neeraj Kumar, Rama Tyagi, Swati Madan, Ramanpreet Walia, Shikha Saxena

**Affiliations:** 1 Department of Pharmacy, Sharda School of Pharmacy, Sharda University, Greater Noida 201310, India; nilanchalasahu24@gmail.com; 2 Department of Pharmacognosy, College of Pharmacy, King Saud University, P.O. Box 2457, Riyadh 11451, Saudi Arabia; 3 Department of Pharmaceutics, School of Pharmaceutical Education and Research, Jamia Hamdard, M. B. Road, New Delhi 110062, India; asad97111@gmail.com; 4 Department of Pharmacognosy & Phytochemistry, School of Pharmaceutical Education and Research, Jamia Hamdard, M. B. Road, New Delhi 110062, India; neerajpaswan225@gmail.com; 5 Amity Institute of Pharmacy, Amity University Uttar Pradesh, Noida 201303, India; tyagirama8@gmail.com (R.T.); smadan3@amity.edu (S.M.); rwalia@amity.edu (R.W.)

**Keywords:** *Solanum xanthocarpum*, transferosomes, topical route, CLSM, dermatokinetic, optimization

## Abstract

The goal of this investigation is to improve the topical delivery of medicine by preparing and maximizing the potential of a nanotransferosome gel infused with *Solanum xanthocarpum* methanolic extract (SXE) to provide localized and regulated distribution. Thin-film hydration was used to create SXE-infused nanotransferosomes (SXE-NTFs), and a Box–Behnken design was used to improve them. Phospholipon 90G (X1), cholesterol (X2) and sodium cholate (X3) were chosen as the independent variables, and their effects on vesicle size (Y1), polydispersity index (PDI) (Y2) and the percentage of entrapment efficiency (EE) (Y3) were observed both individually and in combination. For the SXE-NTFs, the vesicle size was 146.3 nm, the PDI was 0.2594, the EE was 82.24 ± 2.64%, the drug-loading capacity was 8.367 ± 0.07% and the drug release rate was 78.86 ± 5.24%. Comparing the antioxidant activity to conventional ascorbic acid, it was determined to be 83.51 ± 3.27%. Ex vivo permeation tests revealed that the SXE-NTF gel (82.86 ± 2.38%) considerably outperformed the SXE gel (35.28 ± 1.62%) in terms of permeation. In addition, it seemed from the confocal laser scanning microscopy (CLSM) picture of the Wistar rat’s skin that the rhodamine-B-loaded SXE-NTF gel had a higher penetration capability than the control. Dermatokinetic studies showed that the SXE-NTF gel had a better retention capability than the SXE gel. According to the experimental results, the SXE-NTF gel is a promising and successful topical delivery formulation.

## 1. Introduction

The autoimmune skin condition psoriasis affects both the epidermis and dermis and is mediated by T-lymphocytes. Its defining features include epidermal hyperproliferation, keratinocyte hyperplasia and leukocyte infiltration, leading to the formation of psoriatic plaques. Typically, these plaques manifest as crimson regions accompanied by silvery-white scales [1]. Psoriasis is largely influenced by genetic predisposition and aging. Nonetheless, several environmental risk factors have been suggested to play a role in the development of this inflammatory skin condition, including trauma, infection and certain medications [2]. This condition affects around 2–3% of the global population; however, it varies depending on the kind of skin [3]. The management of the condition is determined by its incidence, symptoms and various factors that can impact and exacerbate the disease [4]. It has been logically suggested that treating the affected area locally is essential to avoid harm to nearby tissues, given that this ailment primarily affects skin tissue. Current psoriasis therapies are often time-consuming, ineffective and associated with undesirable side effects. However, for the treatment of mild-to-severe cases, topical drugs, including corticosteroids, coal tar and anthralin, have proven to be quite effective [5]. Hence, there is a crucial need for the development of highly efficient and safe antipsoriatic medications. By addressing the limitations associated with current treatment options, there is significant potential for researching new medications, particularly those derived from natural sources.

*Solanum xanthocarpum* (SX) is an herbal plant from the family *Solanaceae*. In ancient times, the plant was used to treat several skin disorders, and it has wound-healing potency. The ethanolic extract of the stem of SX showed good antipsoriatic activity [6]. Research findings suggest that the methanolic extract of SX (SXE) possesses notable antioxidant properties, indicating its potential to mitigate oxidative stress-related health issues [7]. The extract has shown promise in preclinical studies, demonstrating its ability to modulate different biochemical pathways [8]. Furthermore, the SXE has been explored for its potential in traditional medicine formulations. Its bioactive components are thought to contribute to the plant’s efficacy in promoting respiratory health, managing inflammation and exhibiting antioxidant effects [9].

The exploration of SXE is not merely confined to laboratory investigations. Its traditional use in folk medicine systems underscores its relevance in ethnomedicine and cultural healing practices, emphasizing its potential integration into holistic healthcare approaches that respect and incorporate traditional knowledge. So, to enhance its therapeutic activity, a better formulation is required.

Given that psoriasis is a skin condition and the *Stratum corneum* serves as a barrier to topical administration, employing a method for topical medication delivery proves to be the most successful approach for treating the condition. This strategy not only accelerates the onset of therapeutic benefits but also enhances patient comfort, convenience and compliance [10]. To optimize efficacy, it is advisable to incorporate the medicine into a suitable carrier system.

In this context, the current study aims to create and improve methanolic SX extract-loaded nanotransferosomes (SXE-NTFs) using the independent variables phospholipon 90G (p90G) (X1), cholesterol (X2) and sodium cholate (X3). The enhancement of the transferosome is assessed through the dependent variables vesicle size (Y1), polydispersity index (PDI) (Y2) and entrapment efficiency (EE) (Y3). Additionally, the study evaluates drug release, antioxidant activity (2,2-Diphenyl-1-picrylhydrazyl/DPPH test), skin permeability, confocal laser scanning microscopy (CLSM) and dermatokinetic tests for the enhanced formulation.

## 2. Results and Discussion 

### 2.1. Optimization of SXE-NTF Formulations

**Figure 1:**
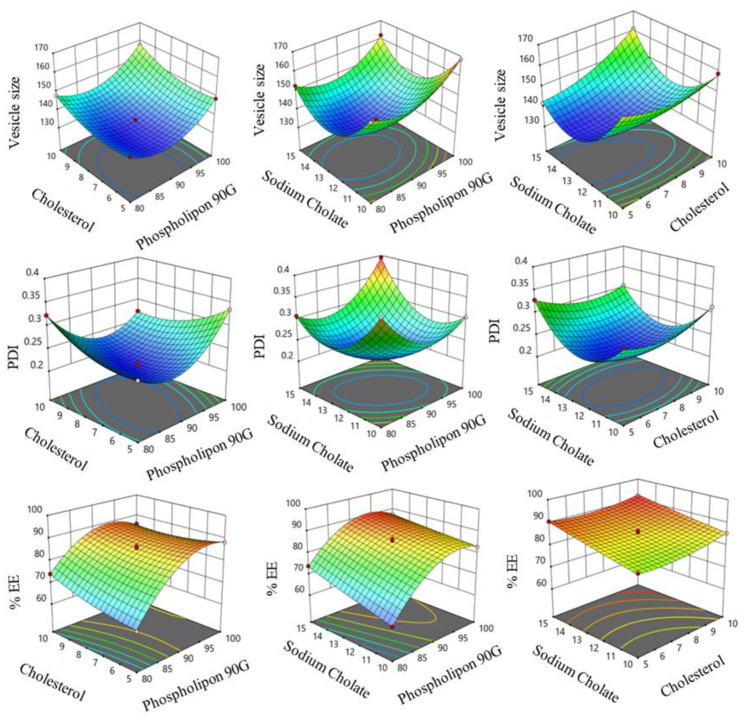
Three-dimensional Box–Behnken design diagrams of vesicle size, PDI and % EE concerning p90G, cholesterol and sodium cholate for 17 formulations.

The 17 formulations generated by the Design Expert software (version 13) were developed, and their outcomes are presented in Table 1. The model summary analysis indicates that the quadratic model was determined to be the most well-conformed model for the chosen three responses, as detailed in Table 2. The impact of independent variables on Y1, Y2 and Y3 (responses) is illustrated in Figure 1. The predicted R^2^ values for this design were found to be within the adjusted R^2^ range.

### 2.2. Response Y1 (Effect of Independent Variables on Vesicle Size)

Table 1 presents the vesicle sizes of the various SXE-loaded formulations, with formulation F16 exhibiting the smallest vesicle size (134.7 nm) and formulation F4 displaying the largest vesicle size (168.43 nm) (Table 1). The inclusion of p90G in the formulation was found to have a favorable impact on the vesicle size. For example, formulation F8, containing 80 mg of p90G, exhibited a vesicle size of 147.8 nm, while formulation F10, with 100 mg of p90G, displayed a vesicle size of 157.73 nm. Similar trends were observed with formulations F13 (80 mg of p90G; vesicle size of 152.64 nm) and F9 (100 mg of p90G; vesicle size of 162.17 nm).

In contrast, the presence of cholesterol in the transferosome had a positive effect on the vesicle size. Increasing the cholesterol content in the formulation was associated with an increase in the vesicle size of the transferosomes. For instance, formulation F1 with 5 mg of cholesterol showed a vesicle size of 141.97 nm, while formulation F12 with 10 mg of cholesterol had a vesicle size of 161.23 nm. Similar trends were observed with F5 (5 mg of cholesterol; vesicle size of 141.09 nm) and F8 (10 mg of cholesterol; vesicle size of 147.8 nm).

However, sodium cholate had a detrimental effect on the vesicle size. Formulation F17, containing 10 mg of sodium cholate, exhibited a vesicle size of 160.18 nm, whereas formulation F1, with 15 mg of sodium cholate, showed a vesicle size of 141.97 nm. Similar findings were observed for F13 (15 mg of sodium cholate; vesicle size of 152.64 nm) and F15 (10 mg of sodium cholate; vesicle size of 157.98 nm) (Figure 1).

### 2.3. Response Y2 (Effect of Independent Variables on PDI)

Formulation F3 exhibited the lowest PDI at 0.2151, while formulation F9 had the highest PDI at 0.3946 (Table 1). The inclusion of p90G in the formulation demonstrated a favorable impact on the PDI. For example, formulation F5, containing 80 mg of p90G, showed a PDI of 0.2594, while formulation F6, with 100 mg of p90G, displayed a PDI of 0.3351. Cholesterol, on the other hand, had a detrimental effect on the PDI; an increase in the cholesterol levels resulted in decreased PDI values. Formulations F1 (5 mg of cholesterol) and F12 (10 mg of cholesterol) had PDIs of 0.3281 and 0.3046, respectively. Interestingly, sodium cholate was found to have a beneficial effect on the PDI. Formulation F4, containing 10 mg of sodium cholate, displayed a PDI of 0.3051, while formulation F9, with 15 mg of sodium cholate, exhibited a PDI of 0.3946 (Figure 1).

### 2.4. Response Y3 (Effect of Independent Variables on % EE)

The lowest percent EE, at 62.04%, was observed in formulation F15, while the highest percent EE, at 92.04%, was found in formulation F12. According to the 3D response graph (Figure 1), the percentage EE increased in the presence of p90G, and the EE of SXE in transferosomes also increased with higher levels of p90G. Formulation F5 (p90G concentration of 80 mg) had an EE of 64.23%, whereas formulation F6 (p90G concentration of 100 mg) demonstrated an EE of 88.24%. Similar trends were observed with formulation F15, containing 80 mg of p90G, and formulation F4, containing 100 mg of p90G, both showing an EE of 82.79% (Table 1).

Additionally, cholesterol had a significant impact on EE; an increase in the cholesterol levels corresponded to an increase in the vesicle-based EE of SXE. In contrast to F7 (cholesterol concentration of 10 mg), which had an EE of 85.39%, F17 (cholesterol concentration of 5 mg) displayed an EE of 81.79%. The EE for formulations F5 (cholesterol concentration of 5 mg) and F8 (cholesterol concentration of 10 mg) were 64.23% and 73.99%, respectively.

When the sodium cholate levels increased, the EE also rose, indicating a positive impact on the EE. In comparison to formulation F1, which included 15 mg of sodium cholate, formulation F17, containing 10 mg of sodium cholate, had an EE of 81.79%, whereas formulation F1’s EE was 90.54%. Similar results were observed for formulations F4 (Sodium cholate: 10 mg) and F9 (Sodium cholate: 15 mg), both showing EEs of 82.79% and 87.45%, respectively (Table 1).

**Figure 2:**
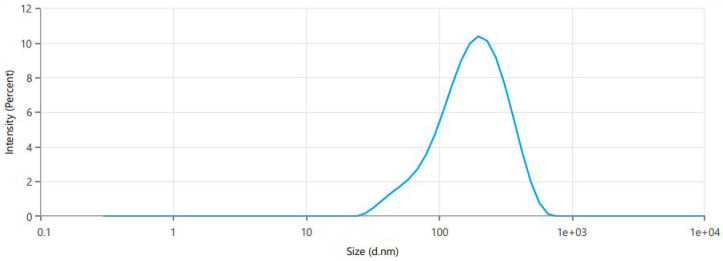
Graph of vesicle size and PDI made using nanozetasizer for optimized SXE-NTF.

**Figure 3:**
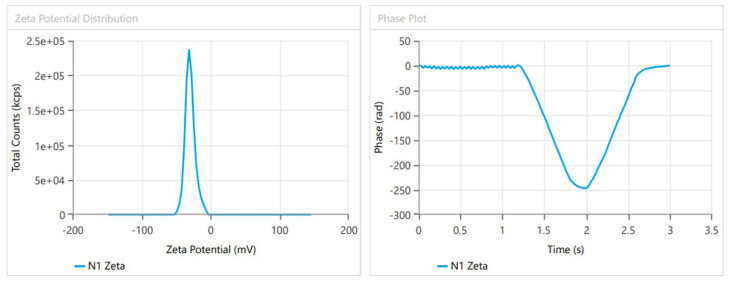
Graph for zeta potential of SXE-NFTs for optimized SXE-NTFs.

Based on the previously provided results, the Design Expert software’s point prediction optimization process led to the creation of the optimal formulation. It was determined that the formulation containing sodium cholate (12.5 mg), cholesterol (7.5 mg) and p90G (90 mg) met the criteria for an optimum transferosome formulation. The Design Expert software predicted values that closely aligned with the optimal formulation’s vesicle size of 146.3 nm (Figure 2), exhibiting a PDI of 0.2596 and an EE of 82.24%. The predicted values were a vesicle size of 135.514 nm, a PDI of 0.21562 and an EE of 85.572%. The zeta potential measurements for the optimized formulation indicated a value of −29.69 mV (Figure 3). Negatively charged vesicle compositions are known to significantly enhance the transdermal administration of treatments through the skin [11].

### 2.5. Vesicle Size and PDI

Through the application of the dynamic light scattering method and a nano-zetasizer, it was determined that the optimized SXE-NFTs exhibited particles with a size of 146.3 nm. The particle size distribution was notably narrow, demonstrating an extremely low PDI of 0.2594 (Figure 2).

### 2.6. Zeta Potential (ZP)

In general, it is thought that a ZP greater than +30 mV or less than −30 mV offers enough repulsion forces to avoid particle aggregation [12]. The colloidal dispersion was guaranteed to be stable for the optimized SXE-NTF formulation, which exhibited a zeta potential of −29.69 mV (Figure 3).

### 2.7. Estimation of Entrapment Efficiency and Drug-Loading (DL) Capacity

The SXE-NTFs’ EE and DL capacity were found to be 82.24 ± 2.64% (n = 3) and 8.367 ± 0.07% (n = 3), respectively, which confirms the maximum amount of SXE entrapped into the vesicle of the nanotransferosomes (NTFs).

### 2.8. Transmission Electron Microscopy Imaging (TEM)

**Figure 4:**
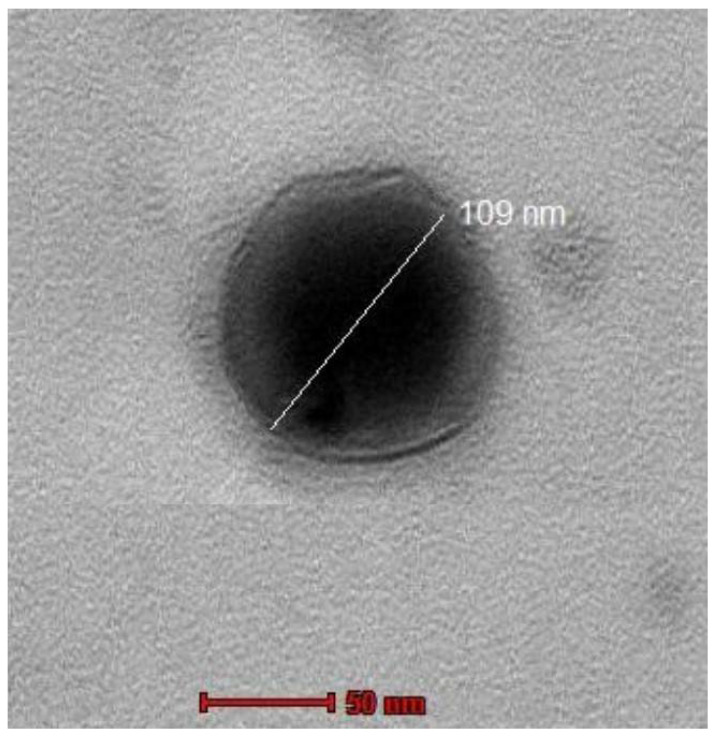
TEM image for optimized SXE-NFTs.

The TEM image of the enhanced formulation revealed spherical shapes and a well-defined sealed structure for the SXE-NFTs. Notably, the size observed in the TEM picture was approximately 109 nm, a characteristic that supports the optimal permeability of the transferosome for transdermal drug administration (Figure 4).

### 2.9. Thermal Analysis with Differential Scanning Calorimeter (DSC)

**Figure 5:**
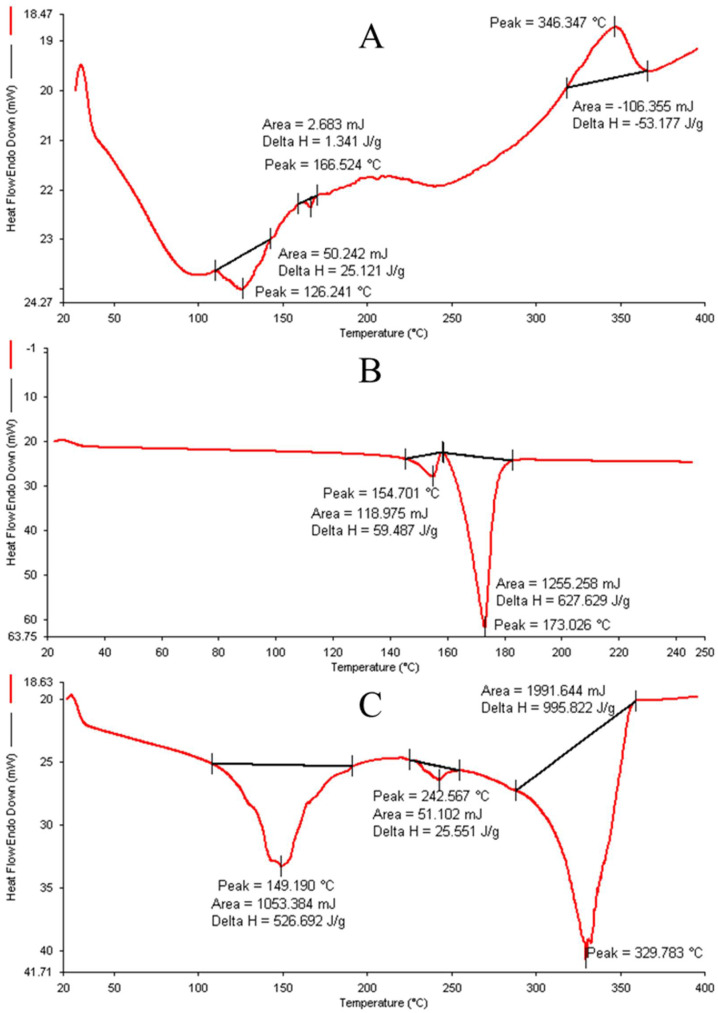
Graphs of DSC ((**A**) SXE, (**B**) lyophilized NFTs and (**C**) lyophilized, optimized SXE-NTFs).

The DSC thermogram (Figure 5) was conducted using the lyophilized, optimized NFTs; SXE; and lyophilized, optimized SXE-NFTs. The data revealed distinct peaks for the lyophilized optimized SXE-NFTs compared to the peaks obtained for SXE alone, as illustrated in the figure. This observation suggests that in the combination of SXE with lyophilized NFTs, no additional free SXE peak was detected, indicating the complete encapsulation of the drug. This confirmation supports the formation of the optimized SXE-NFTs without any predicted leakage of SXE.

### 2.10. Extract and Excipient Compatibility Study Using Fourier Transform Infrared (FTIR) Spectroscopy

**Figure 6:**
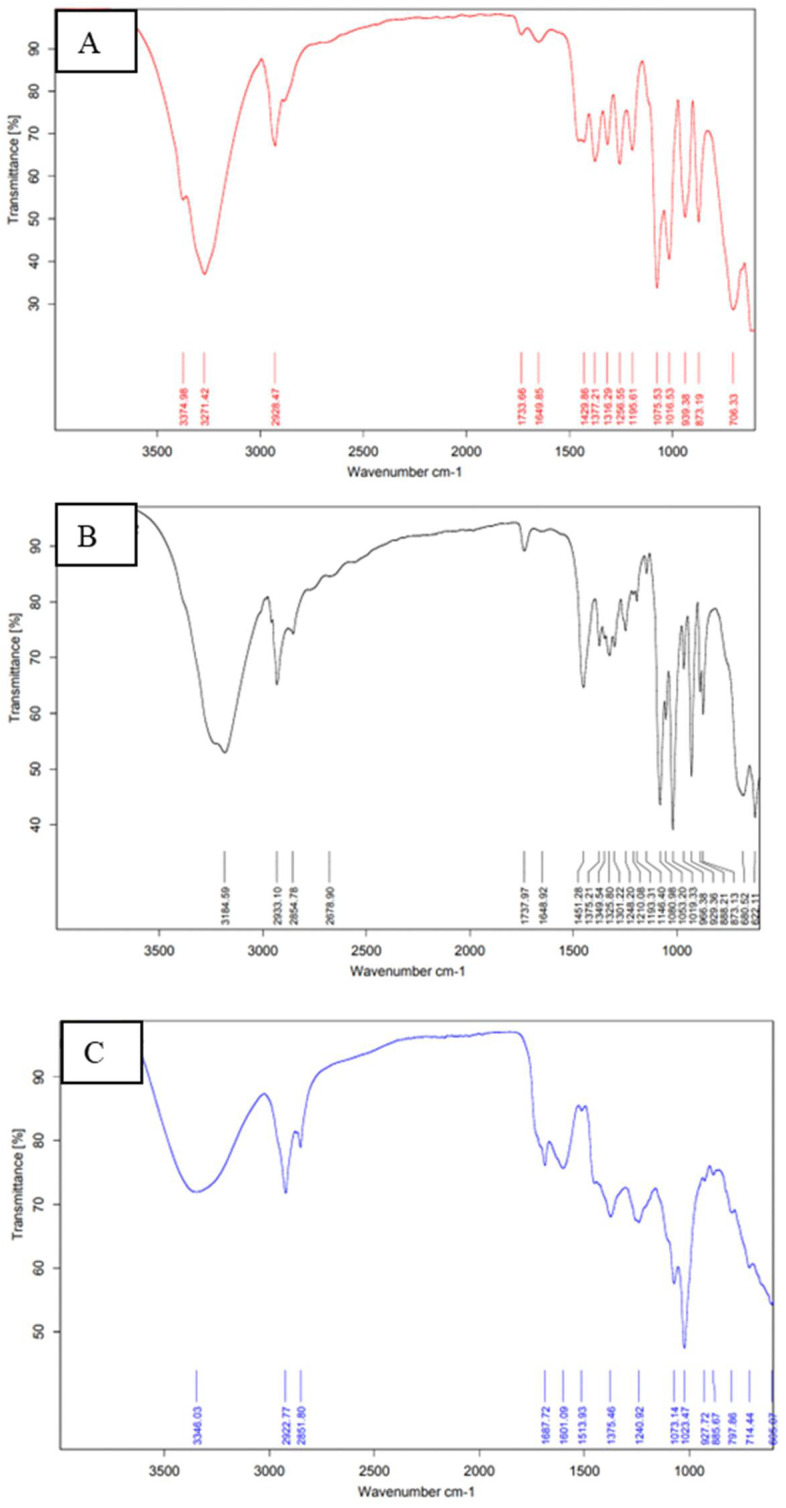
FTIR images for excipient compatibility check ((**A**) lyophilized NFTs, (**B**) lyophilized optimized SXE-NFTs, (**C**) SXE).

The FTIR spectra of the SXE, lyophilized NFTs, and lyophilized, optimized SXE-NFTs are presented in Figure 6. Upon analysis of the mixture of the SXE, lyophilized NFTs and lyophilized optimized SXE-NFTs, no significant differences were observed between the FTIR spectra of the individual drugs and those of their distinctive peaks in the physical mixture. This suggests that there were no discernible chemical interactions between the SXE and the excipients of the NFTs. The observed alterations in the peaks of the SXE-NFTs suggest the entrapment of the SXE within the NFTs. Notably, there is no discernible shift in the peak located between the NTF and SXE-NTF peaks. This absence of change is attributed to the identical functional groups present in both the NTFs and SXE-NTFs, where the latter exhibits combined peaks derived from both the SXE and NTFs.

### 2.11. In Vitro Release Study

**Figure 7:**
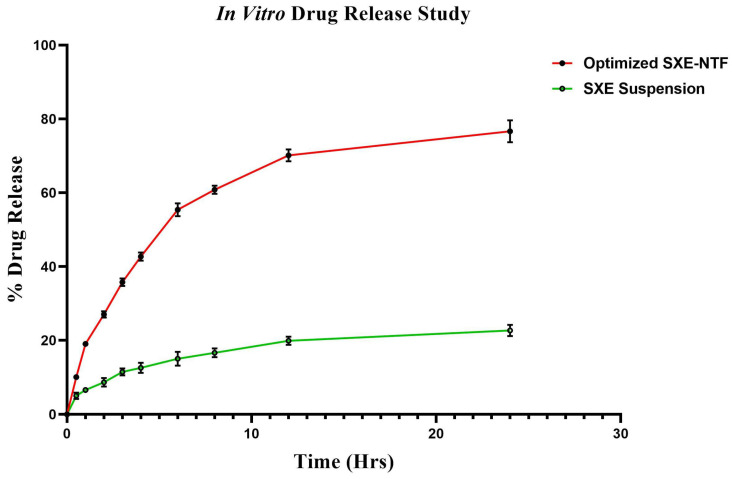
Graph for in vitro drug release study for optimized SXE-NFTs and SXE suspension. The graph was found to be significant (*p* < 0.0001, n = 3), and data are shown as mean ± SD (GraphPad Prism 1.8.0.1., San Diago, CA, USA).

In comparison, the improved formulation for the SXE-NFTS exhibited a release of 78.86 ± 5.24% of the SXE via a dialysis bag, whereas the in vitro SXE release from the SXE suspension across a dialysis bag was reported to be 19.28 ± 1.56% (Figure 7). Various mathematical kinetics models (Korsmeyer–Peppas, first-order, zero-order, Higuchi) were applied to fit the data acquired from the in vitro drug release experiment (Supplementary Figure S1). The release of the SXE from the Korsmeyer–Peppas Model’s optimized SXE-NFTSs displayed the highest correlation coefficient (R^2^) value (R^2^ = 0.9755), followed by Higuchi’s model (R^2^ = 0.9393), the first-order release model (R^2^ = 0.9078) and the zero-order model (R^2^ = 0.7886). After obtaining the highest correlation coefficient value, the enhanced SXE-NFTs determined that the Korsmeyer–Peppas model was the best-fit model (Table 3). A value of n (the release exponent) between 0.5 and 1 (i.e., 0.515) was found by fitting the data to the Korsmeyer–Peppas model to study the release mechanism of the SXE from the optimized SXE-NFTs, indicating that the SXE discharged from the optimized SXE-NFTs follows non-Fickian (anomalous) diffusion.

### 2.12. DPPH Antioxidant Assay

The antioxidant capacity of the SXE-NTF formulation was compared to a standard reference solution (ascorbic acid). The reference solution (ascorbic acid) and the SXE-NTF optimized formulation’s antioxidant activities (% Scavenging) were 89.64 ± 4.89% and 83.51 ± 3.27%, respectively, validating the formulation’s antioxidant capacities.

### 2.13. Characterization and Evaluation of SXE-NTF Gel

**Figure 8:**
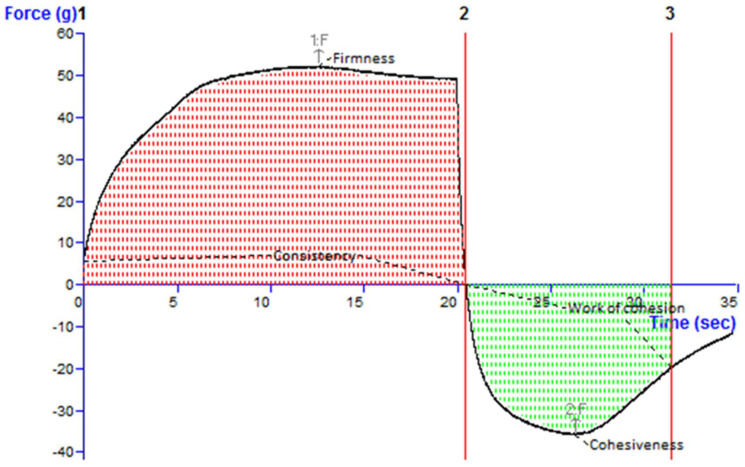
Texture analysis curve plot for optimized SXE-NTF-loaded gel.

Several distinctive qualities of the improved SXE-NTF gel were assessed. The developed gel exhibited a pleasing and uniform appearance, with no visible abrasive particles. The pH of the formulated dermal gel was determined to be 5.82 ± 0.19, rendering it suitable for transdermal application and skin-compatible. Gel spreadability is a crucial factor influencing patient adherence and satisfaction with ongoing application. The study revealed that the extrudability and spreadability of the formulated gel were 161.27 ± 2.74 g and 6.89 ± 0.378 g*cm/s, respectively. The improved SXE-NTF gel formulation displayed a firmness of 52.39 g, a consistency of 915.29 g.s, a cohesiveness of −36.12 g and a viscosity index of −321.67 g.s, as determined by the findings of the texture study (Figure 8).

### 2.14. Ex Vivo Skin Permeation

**Figure 9:**
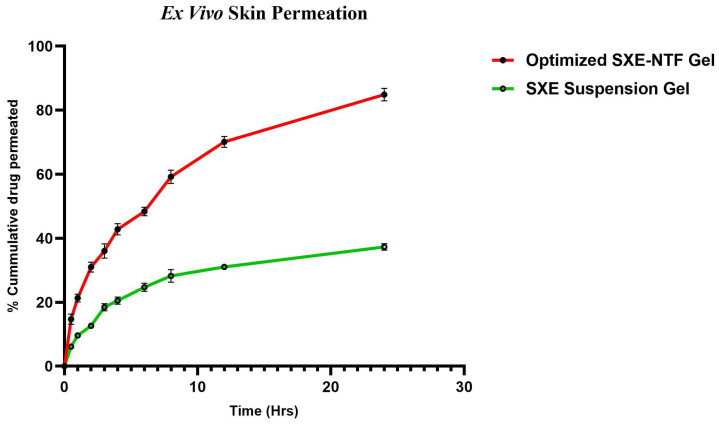
Ex vivo skin permeation graph for the SXE-NTF gel and the SXE gel. The graph was found to be significant (*p* < 0.0001, n = 3), and the data are shown as mean ± SD (GraphPad Prism 1.8.0.1., San Diago, CA, USA).

The ex vivo skin permeation investigation revealed that 35.28 ± 1.62% of the SXE from the SXE-containing gel permeated through the skin. In contrast, the improved SXE-NTF gel formulation demonstrated significantly enhanced penetration, with 82.86 ± 2.38% of the SXE penetrating into the skin of the Wistar rat (Figure 9).

### 2.15. Confocal Laser Scanning Microscopy

**Figure 10:**
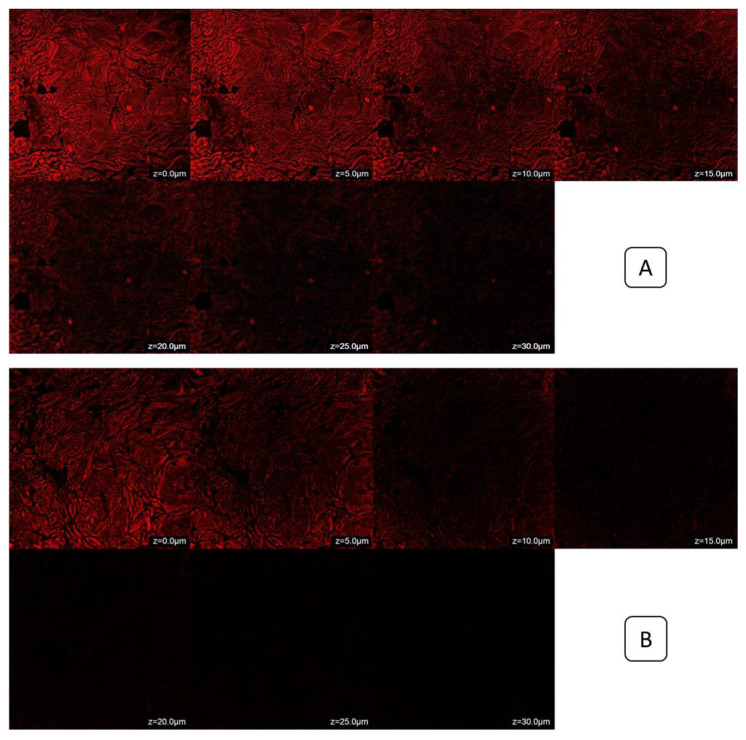
CLSM images ((**A**) Rhodamine-B-loaded SXE-NTF gel, (**B**) Rhodamine-B-loaded gel).

The rhodamine-B-loaded gel exhibited penetration only up to 15.0 μm, indicating that the dye was limited to reaching the top layers of the rat’s skin [Figure 10A]. In contrast, the rhodamine-B-loaded SXE-NTF gel penetrated to a deeper layer, reaching a depth of 30.0 μm [Figure 10B], which the former did not achieve. The formulation may still be present in the lower epidermal layer of the rat’s abdomen skin, as indicated by the stronger fluorescence in that region. This characteristic is desirable for skin diseases affecting the lower epidermal layer. The produced SXE-NTF gel was found to effectively transmit the rhodamine B dye into the deeper layers of the rat’s abdomen skin.

### 2.16. Dermatokinetics

**Figure 11:**
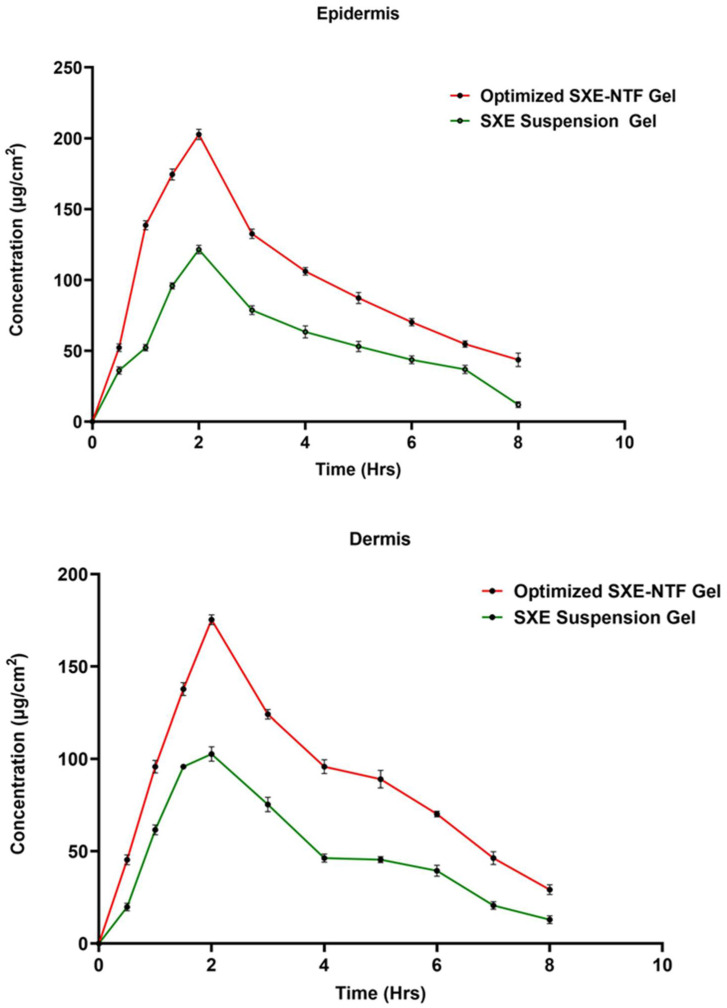
Graphs for dermatokinetics for the epidermis and dermis region of the skin. The graphs were found to be significant (*p* < 0.0001, n = 3), and data are shown as mean ± SD (GraphPad Prism 1.8.0.1., San Diago, CA, USA).

Figure 11 depicts the concentration of SXE in the Wistar rat’s skin dermis and epidermis after the application of the SXE gel and the SXE-NTF gel at predefined time intervals. The dermatokinetic parameter values are presented in Table 4. For the rat skin treated with the SXE gel, the C_Skin max_ values in the epidermis and dermis were 121.4893 ± 6.00 g/cm^2^ and 102.6803 ± 4.00 g/cm^2^, respectively. In comparison, the rat skin treated with the SXE-NTF gel showed higher C_Skin max_ values of 202.7102 ± 11.00 g/cm^2^ in the epidermis and 175.3516 ± 14.00 g/cm^2^ in the dermis.

The AUC_0-t_ values for the rat skin treated with the SXE gel were 399.0724 ± 12.00 g/cm^2^ h in the dermis and 464.9895 ± 10.00 g/cm^2^ h in the epidermis, which are comparable to human skin. Conversely, the rat skin treated with the SXE-NTF gel exhibited higher AUC_0-t_ values of 807.5279 ± 15.00 g/cm^2^ h in the epidermis and 710.875 ± 20.00 g/cm^2^ h in the dermis. The SXE was significantly better retained in both layers of the rat skin after the treatment with the SXE-NTF gel compared to the treatment with the SXE gel formulation. This enhancement may be attributed to the nanosized vesicles formed, facilitating easier penetration into the lipid layers of the skin.

### 2.17. Stability

The results of the short-term accelerated stability experiments were evaluated for three months. No appreciable changes in the particle size, zeta potential, drug content, reconstitution time, color appearance, phase separation, clarity, homogeneity, pH or drug content were found throughout the experiment (Table 5 and Table 6). This study therefore validates the notion that nanoparticles are durable over long periods of storage.

### 2.18. Discussion

In the field of dermatology, psoriasis presents itself as a multifaceted puzzle, a skin condition that goes beyond the surface to delve into the complexities of the immune response and genetic intricacies [13]. Drug delivery in the context of psoriasis encounters hurdles arising from the compromised skin barrier, diverse disease manifestations and the delicate balance required between localized and systemic interventions [14]. Challenges extend to ensuring patient compliance, preserving drug stability in inflamed skin conditions and accommodating individual preferences, posing complexities in the creation of accessible and efficacious treatments [15]. The efficacy of herbal treatments in psoriasis remains a subject of ongoing research, with some studies suggesting potential benefits in symptom relief and inflammation reduction [16]. The medicinal properties of SX include anti-psoriatic, anti-inflammatory, anticancer, immunosuppressive and wound-healing potential [17]. However, due to the size of vesicles in nanometers, their solubility and their improved permeability, extracts have permeation-related concerns that may be minimized by formulating them into a nanoformulation, increasing their bioavailability with pharmacological action, etc. NTFs offer a promising avenue for delivering SXE topically in psoriasis treatment, enhancing skin penetration and bioavailability. Their small size and deformability enable the efficient transport of bioactive compounds, potentially improving the therapeutic efficacy of SXEs against psoriatic lesions [18]. To boost their ex vivo and in vitro potential, this work optimizes the pharmacokinetics of SXE-NTFs by employing several transferosome preparations for the preliminary duration, followed by a laboratory-scale preparation method [19]. In the optimization study, the 17 formulations are interpreted regarding their abundances of p90G, cholesterol and sodium cholate relative to the vesicle size, PDI, and EE of the NTF. From the response of the vesicle size optimization data, p90G and cholesterol were found to have a directly proportional relation with the vesicle size of the SXE-NTF, whereas sodium cholate had an inversely proportional relationship with it. The PDI response for SXE-NTF optimization showed that P90G and sodium cholate had a directly proportional relationship with the PDI, whereas cholesterol had an inversely proportional effect on it. The optimization response for the % EE was found to have a directly proportional relation with p90G, cholesterol and sodium cholate, while it depended on the vesicle size of the SXE-NTF as well. All three responses were found to be similar to those found in some other studies that have been carried out on NTFs or other lipid-based nanoparticles [20]. The optimized formulation produced vesicles with a diameter of 146.3 nm, a PDI of 0.2594, an EE of 82.24 ± 2.64% and a drug release rate of 78.86 ± 5.24% as opposed to the conventional formulation’s vesicles with the same specifications. The surface charge distribution of the SXE-NTF was evaluated to be −29.69 mV, which is good for preventing particle aggregation. The vesicle morphology of the SXE-NTF was found to be in the proper spherical form with a size of 109 nm, which ensures skin permeation enhancement. The thermal analysis data confirmed the complete entrapment of the SXE into the NTF, and the excipient compatibility was confirmed by observing no chemical interaction between the excipients of the NTF and the SXE. Also, the antioxidant activity of the SXE-NTF was found to be 83.51 ± 3.27%, ensuring good antioxidant potential in comparison to the ascorbic acid equivalent. The in vitro drug release study of the optimized formulation, SXE-NTF, initially revealed an intense release but later revealed a slow drug release. This slow release may have been brought on by the release of the SXE, which was loosely bound to the excipients and incorporated completely into the formulation, hence why it was released at a slow pace. The models investigated included the Korsmeyer–Peppas, Higuchi, zero-order and first-order models. The Korsmeyer–Peppas model was the one that suited the data on drug release the best and followed non-Fickian diffusion. Then, the SXE-NTF was incorporated into the gel formulation due to its being the most suitable for transdermal drug delivery to treat psoriasis. The optimized SXE-NTF-loaded gel showed a pH, extrudability, spreadability, firmness, consistency, cohesiveness and viscosity index that were found to be suitable for topical drug delivery, and those were found to be 5.82 ± 0.19, 161.27 ± 2.74 g, 6.89 ± 0.378 g*cm/s, 52.39 g, 915.29 g.s., −36.12 g and −321.67 g.s., respectively. In the ex vivo skin permeation study, the optimized SXE-NTF-loaded gel showed 82.86 ± 2.38% release of the SXE, which was far better than the normal gel of the SXE, and this validated the increase in permeation of the SXE into the Wistar rat skin. The depth of penetration of the SXE-NTF gel into the rat’s skin achieved was up to 30 μm, suggesting penetration into a deeper region of skin layers. The drug retention of the SXE-NTF gel was comparatively high compared to the SXE conventional gel in both the epidermis and dermis layers of the skin, as confirmed by dermatokinetic parameters, which denote the site-specific action of transdermal drug delivery and the increase in the bioavailability of the drug. Lastly, the SXE-NTF and the SXE-NTF-loaded gel were found to be stable for up to 3 months with minute changes, indicating that they can be stored and used for a longer period. In comparison with some other research conducted on herbal nanostructured lipid carrier (NTF) formulations, our study was found to be similar and potent in certain cases in terms of drug delivery toward the treatment of psoriasis [21,22]. It was found that the SXE-NTF had a better vesicle size, PDI, % EE, % DL, drug release rate, permeation capability, dermatokinetics and stability.

## 3. Conclusions

Lipid-based nanoparticle formulations present an appealing alternative to traditional vehicles for enhancing the skin’s absorption of drugs. In this study, transferosomes loaded with SXE were successfully prepared and optimized using the Box–Behnken design. The vesicle size, PDI and % EE were selected as variables to assess among the 17 formulations. The optimized formulation exhibited nanosized vesicles with an EE of 82.24 ± 2.64%. FTIR and DSC clearly demonstrated the complex formation of the SXE and excipients. The in vitro release study initially indicated rapid release, followed by a slower release pattern. The best-fitted model, based on the R^2^ value, was identified as the Korsmeyer–Peppas model. The ex vivo skin permeation, CLSM and dermatokinetic investigations revealed that the improved SXE-NTF formulation exhibited enhanced skin penetration and retention in the rat compared to the control. The results of this study also suggest that the SXE-NTF gel formulation may potentially serve as a useful carrier for transdermal drug delivery in the treatment of psoriasis.

## 4. Materials and Methods

### 4.1. Materials

The *Solanum xanthocarpum* plant was purchased from Global Herbs, Khari Bawori, Delhi, India, and authenticated from CSIR-National Institute of Science Communication and Policy Research (NIScPR/RHMD/Consult/2022/4219-20). “Phospholipon 90G, cholesterol, sodium cholate and disodium hydrogen phosphate, potassium dihydrogen phosphate were procured from SD Fine Chemicals, Mumbai, India”. Chloroform, methanol, Carbopol 934 (LR grade and AR grade) and chlorogenic acid were purchased from Merck, Mumbai, India.

### 4.2. Methods

#### 4.2.1. Extraction Preparation of *Solanum xanthocarpum*

The plant parts of SX were finely powdered, and precisely 50 g of the powdered SX was utilized for extraction using a Soxhlet extraction apparatus (Rama Scientific, Delhi, India). Methanol (200 mL) served as the solvent for extraction at a temperature of 70 °C. The extraction process continued until a clear solvent was obtained in the Syphon tube. Subsequently, the solvent-containing extract was removed and filtered through Whatman filter paper. The resulting solution was then boiled in a water bath to remove the solvent and obtain the crude extract of SX (i.e., SXE) in a semi-solid form, which was stored at 20 °C until it was needed for further study [23].

#### 4.2.2. Method of Preparation of Transferosome

The SXE-loaded transferosome formulations were prepared by the thin-film hydration method with a slight modification, which is otherwise known as Bangham’s method [24]. The ingredients used for the preparation included p90G (lipid), cholesterol, SXE and sodium cholate, which were weighed according to the specified quantities. The experimental procedures involved dissolving the lipid phase (p90G and cholesterol) and SXE in an organic solvent (methanol:chloroform = 1:2). The solvent was then extracted under reduced pressure using rotary evaporation, resulting in the formation of a thin film. Subsequently, the thin film was hydrated in an aqueous medium composed of sodium cholate and double-distilled water. When the thin film was heated above its phase-transition temperature along with the aqueous solution, multilamellar vesicles formed. These vesicles were then downsized using an ultrasonication apparatus (Heilscher UP100H, Teltow, Germany) to create unilamellar vesicles. The formulations were finally stored in a refrigerator at 4 °C until needed [25].

#### 4.2.3. Optimization

A three-factor, three-level Box–Behnken design was used to optimize the SXE-NTF using Design Expert software (Version 12, Stat-Ease, MN, USA) [26]. These data were carefully examined to determine how the different process components [the p90G concentration (X1), cholesterol concentration (X2) and sodium cholate concentration (X3)] influenced the vesicle size (Y1), PDI (Y2) and EE (Y3) of the SXE-NTF (Table 7). These independent factors were varied at low, medium and high concentrations to determine the optimal composition. The experimental design comprised 17 formulation runs (different compositions) with five center points to evaluate the influence of independent factors. Polynomial equations and response surface plots were employed to assess the impact of independent variables on the dependent variables. A polynomial equation provides various models, including the linear and quadratic effects of independent variables on the dependent variables. Among these models, the quadratic model emerged as the most suitable, as the variables used demonstrated both individual and combined influences on the dependent variables.

#### 4.2.4. Method of Preparation of Gel Containing SXE-NTFs

The improved SXE-NTF formulation was converted into a gel formulation to stay on the rat’s skin for longer. A weighed amount of Carbopol 934 (1% *w*/*w*) was taken to manufacture the gel, and it was combined with double-distilled water to create gel dispersion. The overnight storage of this gel dispersion was allocated for full swelling. Later, the aforementioned gel basis was supplemented with other ingredients such as triethanolamine to adjust the pH, polyethylene glycol 400 (15% by weight) as a plasticizer and chlorocresol (0.1%) as a preservative. To create a homogenous gel formulation known as SXE-NTF gel, the optimized SXE-NTF was finally introduced dropwise into this previously created gel based on continuous mixing [27]. The gel was prepared in such a way that 10 g of gel had 200 mg of SXE in it, so the final percentage of SXE incorporated into the SXE-NTF gel was 2% *w*/*w*.

#### 4.2.5. Characterization

##### Vesicle Characterization

The vesicle size, polydispersity index (PDI) and zeta potential (ZP) of the developed SXE-NTF were determined with a particle size analyzer (Malvern Instruments, Worcestershire, UK). The surface charge was measured using the zeta potential, and a stable range was determined to be between −30 and +30. The samples being examined possessed double-distilled water diluted 100 times using a 0.45 μm membrane filter before the investigation [28].

##### Entrapment Efficiency and Drug-Loading Capacity

The ultracentrifugation–filtration technique was used to calculate the % entrapment efficiency (% EE) and % drug loading (% DL) of the SXE-NTF [29]. The prepared SXE-NTFs were filtered before being spun in an ultra-centrifuge (Beckman Coulter, Brea, CA, USA; LE 80) at 25,000 rpm for 30 min at 4 °C. After dilution with methanol, the supernatant was collected, and the quantity of the free drug was determined. The supernatant was then examined using an UltraViolet (UV) spectrophotometer (UV-1601, model/Shimadzu Corp, Kyoto, Japan) at 325 nm (Wavelength of Chlorogenic acid) [30]. The EE was calculated using the formula provided below,

% EE = [(D_int_ − D_sup_)/D_int_] × 100,

where D_int_ is the initial amount of SXE in the nanotransferosomes and D_sup_ is the amount of SXE detected in the supernatant.

DL was calculated using the formula given below,

% DL = (Amount of drug entrapped/Total amount of lipids) × 100

##### Morphological Analysis by TEM

The surface morphology of the optimized SXE-NTFs was examined by TEM (Jeol, JEM-1010, Tokyo, Japan) [31]. A gold coating was applied after a transferosome solution (SXE-NTF: water = 1:10) was sprayed onto a silicon wafer, which was subsequently packed into the microscope after air drying at room temperature. The images were captured at an accelerating voltage of 5 kV.

##### Lyophilization

First, 5% mannitol was added as a cryo-protectant to the optimized SXE-NTF and NTF formulations in separate Petri dishes, spreading and obtaining a larger surface area, with freeze-drying overnight. Then, they were lyophilized (Labfreez Instruments, FD-10-R, Changsha, China) at a condenser temperature of <−60 °C, an ultimate vacuum pressure of 4 Pa and a pump rate of 2 L/s, converting the formulations into solid powdered form [20].

##### Thermal Analysis

To verify the encapsulation of the SXE within the NTFs and characterize the nature of the SXE, we conducted DSC (Perkin Elmer, Pyris 6 DSC, Hopkinton, MA, USA). For the analysis, small quantities of SXE, lyophilized NTFs, and lyophilized, optimized SXE-NTFs were placed in separate aluminum pans and hermetically sealed. The DSC instrument was programmed to operate within a temperature range of 20 °C to 400 °C. The heating rate was set at 10 °C/min, and a nitrogen flow of 60 mL/min was maintained throughout the experiment [32]. For data interpretation, the DSC curves were normalized by sample weight.

##### Excipients Compatibility by FTIR

To determine the compatibility of the extract and the excipient, the FTIR (Brucker Tensor 37, Billerica, MA, USA) spectra of the lyophilized, optimized SXE-NTFs, lyophilized NTFs and the SXE in the 4000–400 cm^−1^ band range were obtained using the KBr pellet method [33].

#### 4.2.6. Drug Release Study

To compare the release from the SXE-NTFs to the SXE suspension, an in vitro drug release study using the dialysis bag technique was conducted [34]. The SXE-NTFs and the SXE suspension were added individually to the previously treated dialysis bags (MW-12 kDa), and then both ends of the bags were knotted. The phosphate buffer, pH = 7.4, was submerged in the dialysis bag holding the samples. The beaker was maintained on a magnetic stirrer with a rotation rate of 100 rpm at 37 °C using a thermostatic control. To keep the sink conditions constant, the discharged samples (2 mL) were collected at various time intervals (0.5, 1, 2, 3, 4, 6, 8 and 12 h) and replaced with the same amount. At 325 nm (the wavelength of chlorogenic acid), the liberated material was UV-spectrophotometrically analyzed. To achieve the average findings, each experiment was carried out in three separate runs. The formula below was used to determine Release%:

Release %= (D_S_/D_T_) ×100

where D_S_ indicates the amount of drug diffused into the receiver medium and D_T_ indicates the total active compound amount in the tested formulation.

#### 4.2.7. Antioxidant Activity

The DPPH standard technique was used to assess the antioxidant capacity of the SXE-NTFs [35]. First, 95 μL of DPPH in methanol and 5 μL of test materials (a variety of extracts and chemicals dissolved in methanol) made up the reaction mixture. Due to the antioxidants’ capacity to donate electrons, the violet DPPH solution becomes colorless at normal temperatures. While the reaction mixture included 300 μM of DPPH, different concentrations of test samples were generated. The resulting reaction mixture was given an hour in a dimly lit area to complete the reaction. The color change is an indication of the sample’s antioxidant potential because of its ability to donate hydrogen. At 517 nm, the material underwent a spectrophotometric analysis. A higher free radical scavenging activity is shown by the reaction mixture having a lower absorbance. Spectrophotometrically, the absorbance at 720 nm of ascorbic acid served as a reference standard. The percentage suppression of the DPPH radical by different extracts was calculated using the method below:

% inhibition = {(A_C_ − A_S_)/A_C_} × 100

where A_C_ is the absorbance of the control and A_S_ is the absorbance of the sample. Percentage scavenging was also evaluated in ascorbic acid equivalence.

#### 4.2.8. Characterization and Evaluation of SXE-NTF Gel

##### pH

A digital pH meter (Mettler Toledo, OH, USA) was used to measure the pH of the prepared gels [36]. In 100 cc of distilled water, 1 g of gel was dissolved and kept for two hours at 4 °C. The results of each formulation were provided as the average of three measurements.

##### Extrudability

The technique used to assess the extrudability of the gel formulations was based on the percentage of gel extruded from the tube when a certain load was applied [37]. The greater the quantity extruded, the better the extrudability. Filling the one-ounce tube with the study’s formulation was carried out in a clean lacquered aluminum tube with a 5 mm nasal tip. A weight of 200 g to the bottom of the tube was applied to release the gel. This experiment used the tip to measure how much gel was extruded through it. It was determined that the greater the gel’s extrudability, the easier it is to apply [38].

##### Spreadability

Gels with minimal spreadability but high consistency are preferred [39]. On a wooden block, a lower slide was attached to a glass slide with one end linked to the weight pan, while the other end was attached to a wooden block. The top slides were then attached with a weight pan. Two glass slides were sandwiched with gel (1 g) and loaded with 1000 g for five minutes to uniformly compress the sample. An addition of weight (80 g) was made to the pan. With the assistance of a piece of thread tied to the hook, the time taken for the top plate to travel 10 cm was recorder. Shorter intervals are more likely to be result in dissemination. The spreadability of the two slides was measured by the amount of time (in seconds) it took to separate them.

$$S=M\times \frac{L}{T}$$

S is the spreadability, M is the weight attached to the higher slide, L is the length of the glass slide and T is the amount of time it takes.

##### Texture Analysis

The texture of the generated SXE-NTF gel was examined using a texture analyzer (T.A. XT Plus Stable Micro System Ltd., Surrey, UK) with an A/BE-d35, BACK EXTRUSION RIG 35 mm DISC probe. The analysis was conducted under standardized conditions, including an auto-set time, an auto-trigger (force) type, a trigger force of 5.0 g, a test speed of 0.50 mm/s and a compression test mode with a 10 mm distance. The gel samples were evaluated at a controlled temperature of 25 °C to assess their consistency and texture properties. Then, 50 g of the optimum formulation (SXE-NTF gel) was put in a 100 mL glass beaker, and the surface was kept as smooth as possible to prevent air bubble entrapment [40].

#### 4.2.9. Ex Vivo Skin Permeation Study

The transdermal permeation tests were conducted to evaluate the transdermal capacity of the SXE-NTF gel compared to the SXE gel (~5 mg SXE, as the control group). The abdominal skin of the Wistar rat from the animal house after scarifying the animal was freshly collected (CPCSEA/IAEC/ICP/2023/04, 3 May 2023). The skin was prepared by using a scalpel for the removal of hairs and subcutaneous fat. A phosphate buffer solution (pH = 7.4) was used to preserve the isolated skin after being washed twice or three times with distilled water. The permeation investigations were conducted in a Franz diffusion cell (Rama Scientific, Delhi, India), which has donor and receiver cells with an effective surface area of 1.5 cm^2^. The fixed prepared skin had its bottom side facing the recipient compartment and its top surface facing the donor compartment. The temperature was maintained at 37 ± 0.5 °C throughout the whole experiment to mimic human body temperature. Each receptor compartment was loaded with 20 mL of phosphate buffer (pH = 7.4) to maintain sink conditions and constantly stirred at 600 rpm with the magnetic stirrer attached beneath the Franz diffusion cells. The SXE-NTF gel and the SXE gel were then evaluated, and 2 mL of each was loaded into the donor region. At the indicated time intervals (0.5, 1, 2, 3, 4, 6, 8 and 12 h), 1 mL of the samples were collected from the receptor phase and substituted with fresh release media to maintain the sink conditions. The samples that had been removed were examined using UV spectrophotometry at 325 nm (the wavelength of chlorogenic acid) [41]. Each experiment was repeated through three different runs to obtain the average results. The cumulative quantity of drug permeated per unit area of skin was graphed against time, and the flux was ascertained by determining the slope of the linear region observed in the resulting curves. The calculation of the apparent permeability coefficient for the SXE-NTF gel was performed by Fick’s first law of diffusion, as mentioned below:

Permeability coefficient = flux(mg/cm^2^/h)/drug concentration in the donor compartment

#### 4.2.10. Confocal Laser Scanning Microscopy (CLSM)

A confocal analysis was performed to evaluate the penetration depth of the gel and contrast it with that of the rhodamine-B-loaded gel after the creation of the rhodamine-B-loaded SXE-NTF gel. To measure the penetration depth, a stacked SXE-NTF gel with rhodamine red B dye was created [20]. The procedure for the study was the same as that for the ex vivo skin permeation research. The rhodamine-B-loaded SXE-NTF gel and the rhodamine-B-loaded gel were each applied to the rat’s abdominal skin in their own Franz diffusion cell. The gel that contained only rhodamine B was utilized as a control sample. After six hours, the treated rat’s abdomen skin was removed, washed with double-distilled water and cut into teeny pieces to fit on a microscopic slide. The slides were analyzed with CLSM (Leica TCS SPE; London, UK) to trace the vesicle permeation in the layers of the rat’s abdomen skin. A 488 nm argon laser beam was used in this study to optically stimulate rhodamine B, and fluorescence emission beyond 532 nm was analyzed. The depth of the SXE-NTFs’ penetration was assessed using CLSM and compared to the control.

#### 4.2.11. Dermatokinetic

The drug concentration in the dermal and epidermal layers of the rat’s skin was determined using the findings of the dermatokinetic studies. In the ex vivo skin penetration study, the SXE-NTF gel was tested on the rat’s skin. In this study, Franz diffusion cell skin samples were collected at 0, 1, 2, 4, 6 and 8 h. The acquired skin samples were washed and kept at 60 °C for 3 min, and then the layers of skin were separated and cut into pieces for the complete extraction of the SXE into methanol. After filtering, the recovered SXE (methanolic extract) was used to determine numerous dermatokinetic parameters as well as to analyze the SXE concentration using UV spectrophotometry at 325 nm (the wavelength of chlorogenic acid) [27].

#### 4.2.12. Stability Study

The momentary expedited stabilities of the gel and the SXE-loaded enhanced NTF formulation was assessed. Lyophilized nanoparticles (SXE-NTF formulation) were transferred to an Eppendorf tube, which was sealed and kept at a temperature of 25 ± 2 °C with a RH of 60 ± 5% in a stability chamber. The vesicle size, PDI, % EE, reconstitution time, color appearance, phase separation, clarity, homogeneity, pH and drug concentration were all monitored once a month in the formulation and the gel [42].

## Figures and Tables

**Table 1:** Observed response in Box–Behnken design for the optimization of SXE-NTF formulations.

Formulation Run	X1 p90G (mg)	X2 Cholesterol (mg)	X3 Sodium Cholate (mg)	Y1 Vesicle Size (nm)	Y2 PDI	Y3 EE (%)
1	90	5	15	141.97	0.3281	90.54
2	90	7.5	12.5	135.76	0.2159	85.19
3	90	7.5	12.5	135.61	0.2151	84.86
4	100	7.5	10	168.43	0.3051	82.79
5	80	5	12.5	141.09	0.2594	64.23
6	100	5	12.5	148.9	0.3351	88.24
7	90	10	10	158.9	0.313	85.39
8	80	10	12.5	147.8	0.3225	73.99
9	100	7.5	15	162.17	0.3946	87.45
10	100	10	12.5	157.73	0.2724	85.91
11	90	7.5	12.5	136.1	0.2157	86.45
12	90	10	15	161.23	0.3046	92.04
13	80	7.5	15	152.64	0.3076	73.86
14	90	7.5	12.5	135.4	0.2157	85.68
15	80	7.5	10	157.98	0.3674	62.04
16	90	7.5	12.5	134.7	0.2157	85.68
17	90	5	10	160.18	0.2915	81.79

Independent variables (X1: p90G in mg, X2: Cholesterol in mg, X3: Sodium cholate in mg), Dependent variables (Y1: Vesicle size in nm, Y2: PDI, Y3: EE in %).

**Table 2:** Regression analysis summary for Y1, Y2, and Y3 answers for fitting to the quadratic model.

Quadratic Model	R^2^	Adjusted R^2^	Predicted R^2^	Std. Dev.	C.V. %	Adequate Precision
Response Y1	0.9978	0.9949	0.9715	0.8237	0.5521	52.5036
Response Y2	1.0000	0.9999	0.9996	0.0005	0.1597	508.9363
Response Y3	0.9978	0.9950	0.9826	0.6080	0.7403	65.3868

Y1: vesicle size (nm); Y2: PDI (%); Y3: % EE; C.V.: coefficient of variation; Std. Dev.: standard deviation.

**Table 3:** Drug release kinetic model for dissolution profile.

	Korsmeyer Peppas Model	First Order Release Model	Zero Order Release Model	Higuchi Model
Correlation coefficient (R^2^)	0.9755	0.9078	0.7886	0.9393
Release exponent (n)	0.5383	−0.0004	0.0004	0.0221
Mathematical equation	Q_t_ = kt^n^	ln Q_t_ = ln Q_0_ + K_1_t	Q_t_ = Q_0_ +K_0_t	Q_t_ = K_H_ √t

Where Q is the amount of the drug released at time t, K is a release rate constant, t is the time and n is the release exponent.

**Table 4:** Dermatokinetic parameters in rat skin after treatment with the SXE gel and the SXE-NTF gel.

	SXE Gel	SXE Gel	SXE-NTF Gel	SXE-NTF Gel
Dermatokinetics Parameters	Epidermis	Dermis	Epidermis	Dermis
Tskin max (h)	2 ± 0.05	2 ± 0.10	2 ± 0.05	2 ± 0.10
Cskin max (μg/cm^2^)	121.48 ± 4.00	102.68 ± 5.00	202.71 ± 9.00	175.35 ± 11.00
AUC_0-t_ (μg/cm^2^h)	464.98 ± 8.00	399.07 ± 11.00	807.52± 14.00	710.87 ± 17.00
Ke (h^−1^)	0.15 ± 0.003	0.15 ± 0.004	0.12 ± 0.005	0.12 ± 0.002

**Table 5:** Short-term accelerated stability evaluation of SXE-NTF formulation.

Evaluation Parameters	Initial	1 Month		2 Months		3 Months	
		4 ± 2 °C	25 ± 2 °C/ 60 ± 5% RH	4 ± 2 °C	25 ± 2 °C/ 60 ± 5% RH	4 ± 2 °C	25 ± 2 °C/ 60 ± 5% RH
Appearance	+++	+++	+++	++	++	++	+
Phase separation	NO	NO	NO	NO	NO	NO	NO
shape	Spherical	Spherical	Spherical	Spherical	Spherical	Spherical	Spherical
Vesicle size (nm)	146.3	146.5	149.8	154.1	155.9	158.5	156.2
PDI	0.2594	0.2596	0.2607	0.2748	0.2761	0.2811	0.2849
EE (%)	82.24	81.66	78.83	79.34	73.71	75.68	70.58
Reconstitution time (s)	10 ± 2	11 ± 2	15 ± 5	10 ± 3	16 ± 4	14 ± 3	20 ± 4

+ satisfactory; ++ good; +++ excellent; relative humidity—RH.

**Table 6:** Short-term accelerated stability evaluation of the SXE-NTF gel formulation.

Evaluation Parameters	Initial	1 Month		2 Months		3 Months	
		4 ± 2 °C	25 ± 2 °C/ 60 ± 5% RH	4 ± 2 °C	25 ± 2 °C/ 60 ± 5% RH	4 ± 2 °C	25 ± 2 °C/ 60 ± 5% RH
Color	Slightly Brownish	Slightly Brownish	Slightly Brownish	Slightly Brownish	Slightly Brownish	Slightly Brownish	Slightly Brownish
Appearance	Translucent	Translucent	Translucent	Translucent	Translucent	Translucent	Translucent
Phase Separation	NO	NO	NO	NO	NO	NO	NO
Clarity	YES	YES	YES	YES	YES	YES	YES
pH	5.82 ± 0.19	5.83 ± 0.05	5.84 ± 0.08	5.86 ± 0.12	5.81 ± 0.06	5.85 ± 0.14	5.91 ± 0.08
Homogeneity	***	***	**	***	**	**	*
Washability	Washable	Washable	Washable	Washable	Washable	Washable	Washable
Odor	NO	NO	NO	NO	NO	NO	NO

* satisfactory, ** good, *** excellent.

**Table 7:** Level of independent variables used to optimize SXE nanotransferosome (SXE-NTF) with Box–Behnken design.

Variables			
**Independent variables**	**Low**	**Medium**	**High**
X1 = p90G (mg)	80	90	100
X2 = Cholesterol (mg)	5	7.5	10
X3 = Sodium cholate (mg)	10	12.5	15
**Dependent variables**			
Y1 = Vesicle size (nm)			
Y2 = PDI			
Y3 = EE (%)			

## Data Availability

The data presented in this study are openly available in article.

## Supplementary Materials

The following supporting information can be downloaded at https://www.mdpi.com/article/10.3390/gels10020119/s1: Figure S1: Drug release kinetic model diagrams of Korsmeyer Peppas Model, First Order Release Model, Zero Order Release Model, and Higuchi Model for SXE-NTF formulation; Figure S2: Animal Approval Form; Content: Regression equation of the fitted quadratic model.
