# Supplementary material for: Optimization, In Vitro and Ex Vivo Assessment of Nanotransferosome Gels Infused with a Methanolic Extract of Solanum xanthocarpum for the Topical Treatment of Psoriasis
Source: Gels. 2024 Feb 2;10(2):119. doi: 10.3390/gels10020119 (PMC10888226; doi:10.3390/gels10020119)
Supplement: Supplementary file 1 [file gels-10-00119-s001.zip › gels-2808859-supplementary.pdf]

# <u>Supplementary file</u>

# Optimization, In Vitro and Ex Vivo Assessment of Nanotransferosome Gels Infused with a Methanolic Extract of *Solanum xanthocarpum* for the Topical Treatment of Psoriasis

**Nilanchala Sahu [1], Perwez Alam [2,*], Asad Ali [3], Neeraj Kumar [4], Rama Tyagi [5], Swati Madan [5], Ramanpreet Walia [5] and Shikha Saxena [5,*]**

[1] Department of Pharmacy, Sharda School of Pharmacy, Sharda University, Greater Noida 201310, India; nilanchalasahu24@gmail.com
[2] Department of Pharmacognosy, College of Pharmacy, King Saud University, P.O. Box 2457, Riyadh 11451, Saudi Arabia
[3] Department of Pharmaceutics, School of Pharmaceutical Education and Research, Jamia Hamdard, M. B. Road, New Delhi 110062, India; asad97111@gmail.com
[4] Department of Pharmacognosy & Phytochemistry, School of Pharmaceutical Education and Research, Jamia Hamdard, M. B. Road, New Delhi 110062, India; neerajpaswan225@gmail.com
[5] Amity Institute of Pharmacy, Amity University Uttar Pradesh, Noida 201303, India; tyagirama8@gmail.com (R.T.); smadan3@amity.edu (S.M.); rwalia@amity.edu (R.W.)
* Correspondence: aperwez@ksu.edu.sa (P.A.); skataria@amity.edu (S.S.)

*Regression equation of the fitted quadratic model:*

Vesicle size = + 135.51 + 4.72 × $X_1$ + 4.19× $X_2$ - 3.44 × $X_3$ + 0.5300 × $X_1$ × $X_2$ - 2.300× $X_1$ × $X_3$ + 5.13 × $X_2$ × $X_3$ + 9.05 × $X_1{}^2$ + 4.32 × $X_2{}^2$ + 15.74 × $X_3{}^2$

PDI = + 0.2156 + 0.0063 × $X_1$ - 0.0002 × $X_2$ + 0.0072 × $X_3$ - 0.0315 × $X_1$ × $X_2$ + 0.0373 × $X_1$ × $X_3$ - 0.0113 × $X_2$ × $X_3$ + 0.0581 × $X_1{}^2$ + 0.0237 × $X_2{}^2$ + 0.0700 $X_3{}^2$

EE = - 913.41425 + 19.22618 × $X_1$ + 8.44690 × $X_2$ + 8.04700 × $X_3$ - 0.120900 × $X_1$ × $X_2$ - 0.071600 × $X_1$ × $X_3$ - 0.084000 × $X_2$ × $X_3$ - 0.091923 × $X_1{}^2$ + 0.274040 × $X_2{}^2$ + 0.024840 $X_3{}^2$

$X_1$: p90G (mg), $X_2$: Cholesterol (mg), $X_3$: Sodium cholate (mg)

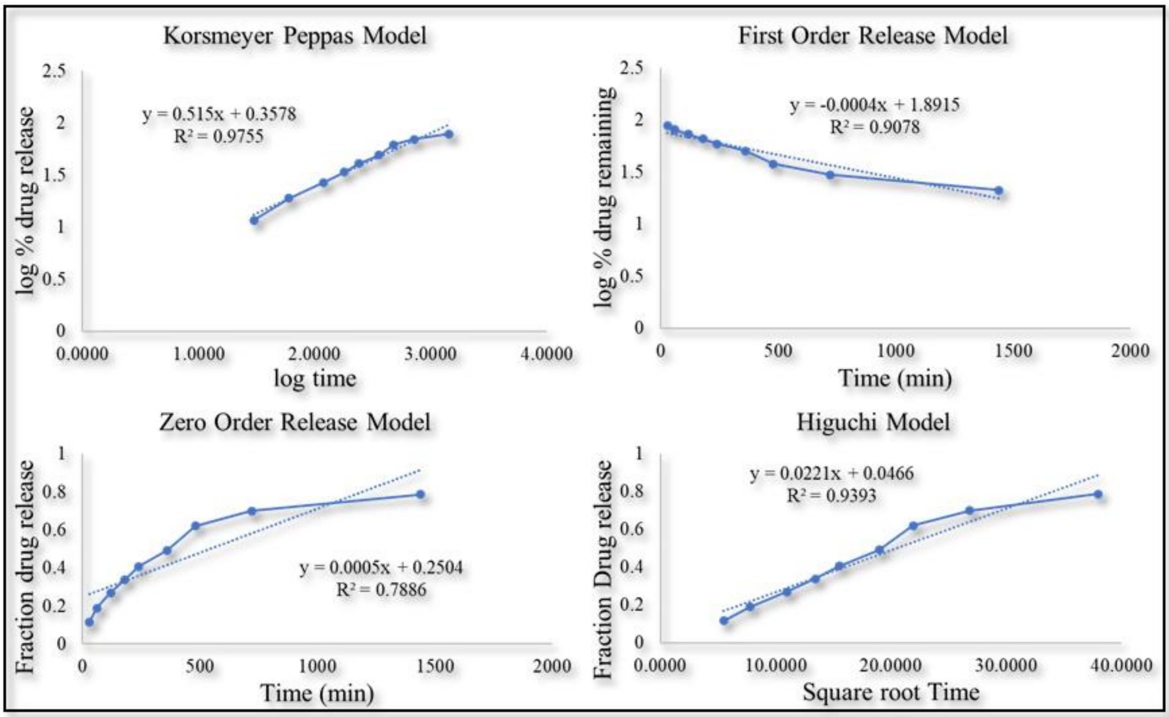


**Figure S1.** Drug release kinetic model diagrams of Korsmeyer Peppas Model, First Order Release Model, Zero Order Release Model, and Higuchi Model for SXE-NTF formulation.

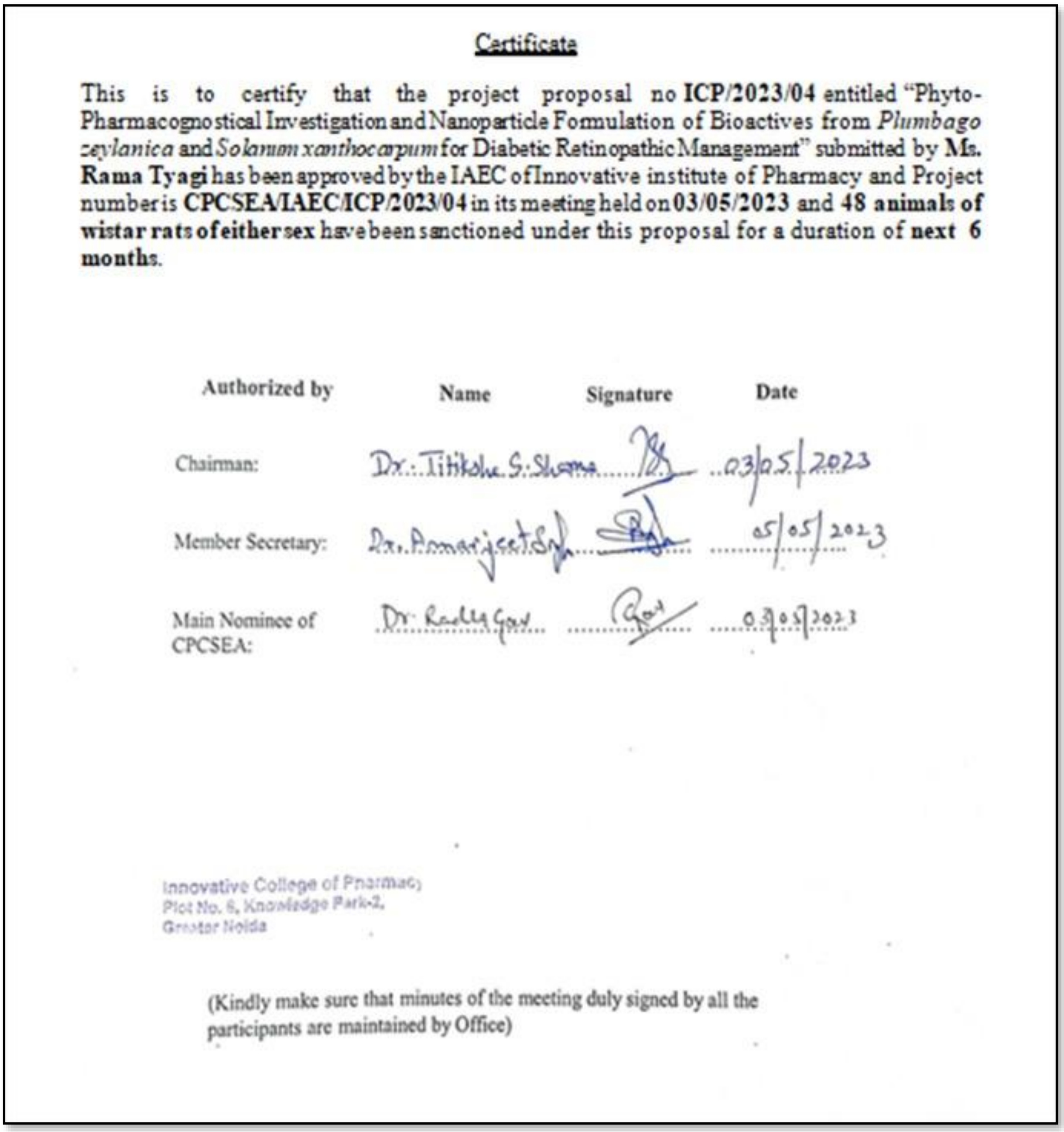

<u>Certificate</u>

This is to certify that the project proposal no **ICP/2023/04** entitled "Phyto-Pharmacognostical Investigation and Nanoparticle Formulation of Bioactives from *Plumbago zeylanica* and *Solanum xanthocarpum* for Diabetic Retinopathic Management" submitted by **Ms. Rama Tyagi** has been approved by the IAEC of Innovative institute of Pharmacy and Project number is **CPCSEA/IAEC/ICP/2023/04** in its meeting held on **03/05/2023** and **48 animals of wistar rats of either sex** have been sanctioned under this proposal for a duration of **next 6 months**.

| Authorized by | Name | Signature | Date |
|---|---|---|---|
| Chairman: | Dr. Titiksha S. Sharma | [illegible] | 03/05/2023 |
| Member Secretary: | Dr. Amarjeet Singh | [illegible] | 05/05/2023 |
| Main Nominee of CPCSEA: | Dr. Radha Goel | [illegible] | 03/05/2023 |

Innovative College of Pharmacy
Plot No. 6, Knowledge Park-2,
Greater Noida

(Kindly make sure that minutes of the meeting duly signed by all the participants are maintained by Office)

**Figure S2**. Animal Approval Form.
